# Giant parieto-occipital encephaloceles: always a dismal prognosis? A case report and review of the literature

**DOI:** 10.1007/s00381-026-07212-0

**Published:** 2026-03-22

**Authors:** Wouter J. Dronkers, Irene M. J. Mathijssen, Attie Go, Victor Volovici, Jochem K. H. Spoor

**Affiliations:** 1 Department of Neurosurgery, Erasmus Medical Center Rotterdam, Doctor Molewaterplein 40, 3015 GD Rotterdam, the Netherlands; 2 Center for Complex Microvascular Surgery, Erasmus MC University Medical Center, Rotterdam, the Netherlands; 3 Sophia Children’s Hospital, Erasmus MC University Medical Center, Doctor Molewaterplein 40, 3015 GD Rotterdam, the Netherlands; 4 Department of Plastic- Reconstructive and Hand Surgery, Erasmus Medical Center Rotterdam, Rotterdam, the Netherlands; 5 Department of Obstetrics and Gynecology, Erasmus Medical Center a Rotterdam, Rotterdam, the Netherlands

**Keywords:** Giant parieto-occipital encephaloceles, Dismal prognosis, Vene of Galen

## Abstract

The presented case involves an infant with a prenatally diagnosed giant encephalocele including parts of the occipital and superior parietal lobes, as well as the superior cerebellum. Imaging revealed a complex encephalocele with absence or hypoplastic aspects of venous structures, including the vein of Galen and transverse and sigmoid sinus. Clinically, despite the anticipated poor prognosis, the patient remained stable after delivery, leading to a reassessment of the initial palliative care plan. Surgical reconstruction of the encephalocele was performed at 4 weeks of age. The most recent follow-up was conducted at 4 years of age. The patient demonstrated significant developmental progress including speech, language, and motor skills without complaints of headache or nausea. The presented case highlights the medical and ethical challenges physicians face while dealing with pediatric patients with severe congenital anomalies. The case emphasizes the importance of personalized care and reconsideration of long-term expectations for these conditions.

## Case report

An encephalocele refers to a severe congenital neurological condition which is characterized by herniation of neural tissue through a defect of the neurocranium. Encephalocele, which is considered a neural tube defect, has an estimated prevalence of 1–5 per 10,000 livebirths [1]. Classification of encephalocele is based on the anatomical location of the skull defect. Most often, the encephalocele is located occipitally [2]. In cases in which the encephalocele is larger than the patient’s head, it is referred to as “giant encephalocele” [3].

Prognosis in patients with a giant encephalocele is largely related to the location, size of the cele, amount of brain tissue affected, and presence of other intra- and extracranial anomalies (Table 1) [2, 4]. Although mortality rates are relatively low in patients with giant occipital encephalocele, various developmental issues exist (Table 2). Delayed milestones are frequently reported ranging from mild cognitive impairment to severe retardation. Motor function including being able to sit or walk without assistance is important yet infrequently reported outcomes. Therefore, adequate counseling of parents and treatment of these patients remain challenging from a medical and ethical point of view.

**Table 1:** Demographic and radiological characteristics

Author	Patients (female)	Sac, dimensions	Sac, contents
Datta et al. [5]	4 (1)	10 × 14 cm–18 × 20 cm	Cystic with small amount of neural tissue (*n* = 3); significant amount of neural tissue (*n* = 1)
Atallah et al. [6]	1 (1)	40 cm circumference	Cystic with small amount of neural tissue
Qudsieh et al. [3]	7 (5)	12 × 10 × 9 cm – 15 × 12 × 10 cm	Not individually specified
Deora et al. [7]	1 (1)	98 cm circumference	Cystic with small amount of neural tissue
Singh et al. [8]	11 (5)	Not specified	Not individually specified
Murthy et al. [9]	1 (1)	20 × 15 × 28 cm	Cystic with small amount of neural tissue
Naik et al. [10]	1 (1)	26 × 34 × 30 cm	Cystic with small amount of neural tissue
Kanesen et al. [11]	1 (1)	44 cm circumference	Cystic with small amount of neural tissue
Ozdemir et al. [12]	4 (4)	14 × 18 cm–20 × 24 cm	Significant amount of neural tissue (*n* = 3); cystic without neural tissue (*n* = 1)
Canaz et al. [13]	1 (0)	9 × 8 cm and 10 × 9 cm*	Significant amount of neural tissue
Mahapatra et al. [14]	13 (NS)**	NS	Not individually specified
Sather et al. [15]	1 (1)	19 × 9 cm	Cystic with small amount of neural tissue
Andarabi et al. [16]	1 (0)	38–60 cm circumference	Cystic with small amount of neural tissue
Mohanty et al. [17]	2 (NS)	25 × 12 cm–30 × 25 cm	Significant amount of neural tissue (*n* = 2)
Mardzuki et al. [18]	3 (2)	22 × 8 cm–28 × 15 cm	Cystic with small amount of neural tissue (*n* = 2); not specified (*n* = 1)
Neupane et al. [19]	1 (0)	20 × 15 × 17 cm	Cystic with small amount of neural tissue

*NS *not specified, *cm *centimeter

*The reported patient had two encephaloceles

**Only patients with occipital encephaloceles were collected

**Table 2:** Shunt dependency, outcomes, and mortality

Author	FU time	Shunt	Outcome	Mortality
Datta et al. [5]	1–13 years	2	Age-appropriate milestones (*n* = 2) Delayed milestones (*n* = 2)	1
Atallah et al. [6]	40 months	1	Delayed milestones (*n* = 1)	0
Qudsieh et al. [3]	2 years	0	Delayed milestones in speech and walking (number of patients not specified)	1
Deora et al. [7]	1 year	1	Age-appropriate milestones; able to walk and crawl; able to track objects	0
Singh et al. [8]	6 months – 4 years	4	Delayed milestones (*n* = 2); doing well (*n* = 2); lost to follow-up (*n* = 2); not specified (*n* = 2)	3
Murthy et al. [9]	2 years	1	Delayed milestones	0
Naik et al. [10]	1 year	1	Age-appropriate milestones; able to hold head; sit without assistance	0
Ozdemir et al. [12]	18–40 months	0	Normal motor & mental development (*n* = 2); motor & mental retardation (*n* = 2)	0
Canaz et al. [13]	3 years	0	Delayed milestones; walking without assistance; equal use of all extremities	0
Mahapatra et al. [14]	6 months – 4 years	5	Age-appropriate milestones (*n* = 9); delayed milestones (*n* = 2)	2
Sather et al. [15]	2 years	1	Delayed milestones; walking without assistance; equal use of all extremities; few word vocabulary	0
Andarabi et al. [16]	2 years	0	Age-appropriate milestones	0
Mohanty et al. [17]	2 years	1	Delayed milestones (*n* = 1); not specified (*n* = 1)	0
Mardzuki et al. [18]	3 years	1	Doing well (*n* = 2); cerebral palsy (*n* = 1)	0
Neupane et al. [19]	NS	1	Good state of health	0

*NS*, not specified

The presented case involved an infant with a prenatally diagnosed severe encephalocele involving herniation of the occipital and superior parietal lobes and superior cerebellum, delivered within our tertiary referral center.

In the presented case, the parents had been counseled by colleagues from the neonatology department before and after delivery regarding the assumed poor prognosis, and the pediatric palliative care team was engaged. The patient was discharged at one week of age with a plan for best supportive, palliative care. In the subsequent days, the patient’s clinical condition remained stable. A reassessment of the initial management plan was undertaken within the multidisciplinary boards, and the patient was readmitted at three weeks of age for further diagnostic evaluation and treatment.

**Fig. 1:**
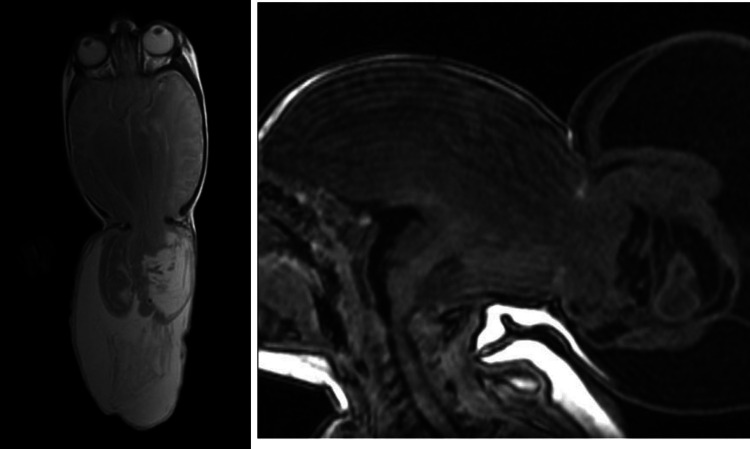
Preoperative postnatal MRI provides an axial and sagittal view of the preoperative MRI showing herniating neural tissue

**Fig. 2:**
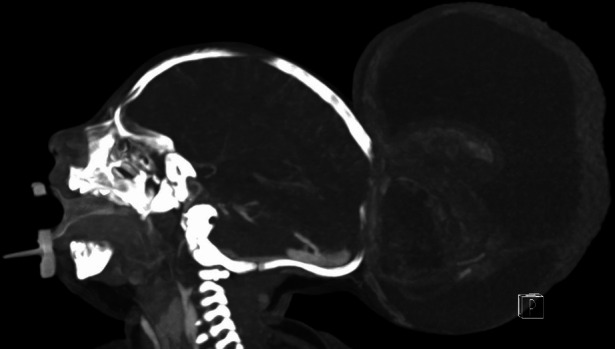
Preoperative CT-angiography reveals the absence of a vein of Galen complex and a hypoplastic transverse and sigmoid sinus on the left side

**Fig. 3:**
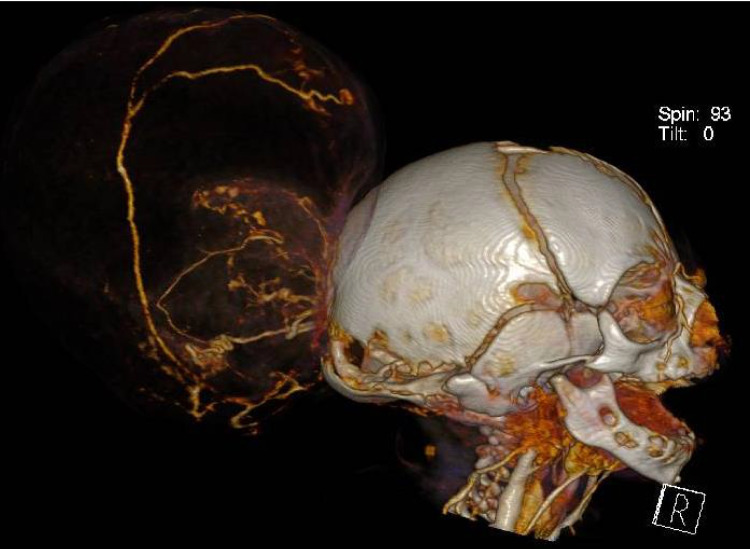
Preoperative CT-angiography

Imaging studies (Fig. 1) demonstrated a severe meningoencephalocele with herniation of both the occipital lobes as well as part of the parietal lobes and the superior cerebellar hemispheres and vermis. Additional CT-angiography (Figs. 2 and 3) identified the confluence of sinuses at the level of the skull defect, with the sinus rectus located within the encephalocele. Notably, imaging also revealed the absence of the vein of Galen and a hypoplastic left transverse and sigmoid sinuses.

**Fig. 4:**
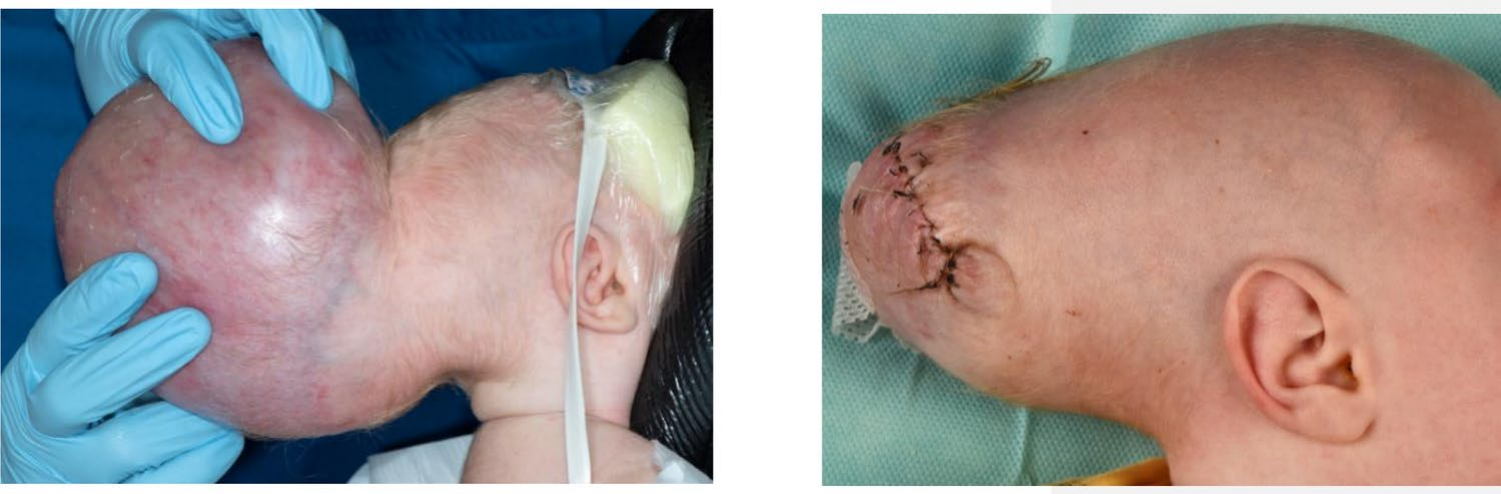
Pre and postoperative clinical photography represents the preoperative (left picture) and postoperative (right picture)

**Fig. 5:**
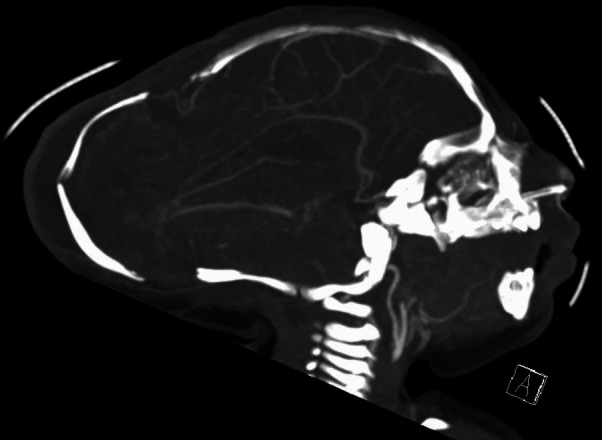
Postoperative CT-angiography, sagittal view

Reconstruction of the encephalocele and skull defect was performed at four weeks of age, together with the Plastic- and Reconstructive Surgery service. The meningocele was opened and the edges of the dura mater under the bone were exposed. Care was taken to not injure the exposed venous outflow. Necrotic tissue was removed from the occipital lobes. These were noted to be smooth, without gyral imprint and without M4 and P4 vessels running on their surface with the use of ultrasound. After excision, the remaining vital brain tissue was covered with a dural substitute. Strips of bone were cut from the skull surrounding the defect and fixated in a fashion covering the maximal surface of the defect with minimal cranial expansion. The extra skin was removed and the cele was closed (Figs. 4 and 5). The procedure was performed with less than 250-mL blood loss. The postoperative phase was uneventful.

The most recent follow-up was conducted at 4 years of age. At this time, significant progress in language, speech, and motor skill development (e.g., being able to walk without assistance and grasp and hold items using both hands) was observed. The patient is able to form up to seven-word sentences and count up to 30. Notably, the patient exhibited no complaints of headache, nausea, or vomiting and imaging studies show development of the parts of the brain not involved in the encephalocele. However, assessment of visual function reveals impairments in vision and color perception.

The case presented demonstrates a complex giant meningoencephalocele, with aberrant venous anatomy, with absence of the vein of Galen and hypoplastic transverse/sigmoid sinuses, but with a relatively less severe clinical presentation than imaging studies and scientific literature suggest. The subsequent evolution might challenge the paradigm to generalize poor outcome to all patients with complex giant encephaloceles.

## Data Availability

No datasets were generated or analyzed during the current study.
